# Lipomatous hypertrophy of the atrial septum – a benign heart anomaly causing unexpected surgical problems: a case report

**DOI:** 10.1186/s12872-018-0892-3

**Published:** 2018-07-28

**Authors:** Grzegorz Bielicki, Marceli Lukaszewski, Kinga Kosiorowska, Jacek Jakubaszko, Rafal Nowicki, Marek Jasinski

**Affiliations:** 1 Department of Cardiac Surgery, Wroclaw Medical University, Wroclaw, Poland; 2 Department of Anaesthesiology and Intensive Therapy, Wroclaw Medical University, Borowska 213, 50-556 Wroclaw, Wroclaw, Poland

**Keywords:** Lipomatous hypertrophy, Interatrial septum, TEE, Computered tomography

## Abstract

**Background:**

Lipomatous hypertrophy of the atrial septum (LHAS) is an anomaly of the heart. It is characterized by an infiltration of adipocytes into myocytes of the interatrial septum, sparing the fossa ovalis, which gives a characteristic hourglass-shaped image. Due to the progress in imaging techniques, it can be recognized more frequently, but it is still often misdiagnosed.

**Case presentation:**

We present a case of 65-year-old woman with an incidentally discovered lipomatous hypertrophy of the atrial septum during cardiac surgery, which has caused the technical problems for surgeons with bicaval cannulation and visualization of the operated structures of the heart. Due to the unclear shadow in the lung parenchyma, the patient had preoperative computed tomography (CT) done, but the study report focused only on the lung description, neglecting visible changes in the structure of the heart. Based on the standardly performed intra-operative transesophageal echocardiography (TEE), as well as by analyzing the chest X-ray and CT scans, the diagnosis of LHAS was made. It allowed the surgeon to leave the mass intact, thus not increasing the risk of the baseline surgery.

**Conclusions:**

LHAS is a rare but increasingly recognized anomaly of the heart. Contemporary diagnostic methods allow to diagnose and make the right therapeutic decisions. The utility of TEE and analysis of X-ray images, in this case, allowed the surgeon to recognize LHAS, and because of its histologically benign nature and asymptomatic course, to leave this change intact. Surgical treatment should be limited only to cases of patients with life-threatening cardiovascular complications.

## Background

Heart tumors are often underestimated by professionals in the field. Being relatively rare, secondary neoplasms are mostly the result of advanced malignant melanoma, lymphoma, and leukemia. Benign tumors such as myxomas, lipomas, papillary fibroelastomas, angiomas, and fibromas represent about 75% of primary cardiac tumors [1, 2]. A separate non-neoplastic benign cardiac lesion, that may be mistaken for various heart tumors, is lipomatous hypertrophy of the atrial septum (LHAS), first described by Prior in 1964, based on autopsy study [3]. The etiology of LHAS has not been recognized. It is presumed that due to the involvement of embryonic mesenchymal cells in the primary formation of atria, the atrial septum cells may differentiate into adipocytes with appropriate stimuli [4]. In the histological image analysys, no mitoses are observed, hence the change does not represent a malignancy [5]. Morphologically, it is presented as a non-encapsulated excessive epicardial fat deposition in the septum secundum, that infiltrates the area of the interatrial septum that spares the fossa ovalis [6]. The thickness of the septum can reach 20 mm and more [7]. Accumulation of adipose tissue can also be observed in subepicardium, crista terminalis, endocardium, and mediastinum. In differential diagnostics, it is required to take into account possible adipose tissue neoplasms. Unlike LHAS, lipomas are encapsulated, round, homogeneous and do not infiltrate myocardium fibers. Lipomatous hypertrophy is associated with obesity and is seen more frequently in elderly and female patients [8, 9]. Development of imaging techniques has enabled more frequent recognition of usual asymptomatic masses [10]. In a prospective study using computed tomography, lipomatous hypertrophy was identified in 2.2% of the patients [11]. Extremely rare infiltration of adipocytes, causing distortion of the septum, may pose life-threatening cardiovascular complications requiring urgent cardiac surgery intervention [12]. The rare complications of LHAS include superior vena cava syndrome, severe cardiac arrhythmias (sick sinus syndrome, arrhythmias, changes in P waveform morphology in ECG), pericardial effusion, heart failure and sudden cardiac death [13, 14]. However, the majority of cases are clinically silent and are detected accidentally during routine chest X-ray, echocardiography, surgery or autopsy [15].

## Case presentation

**Fig. 1:**
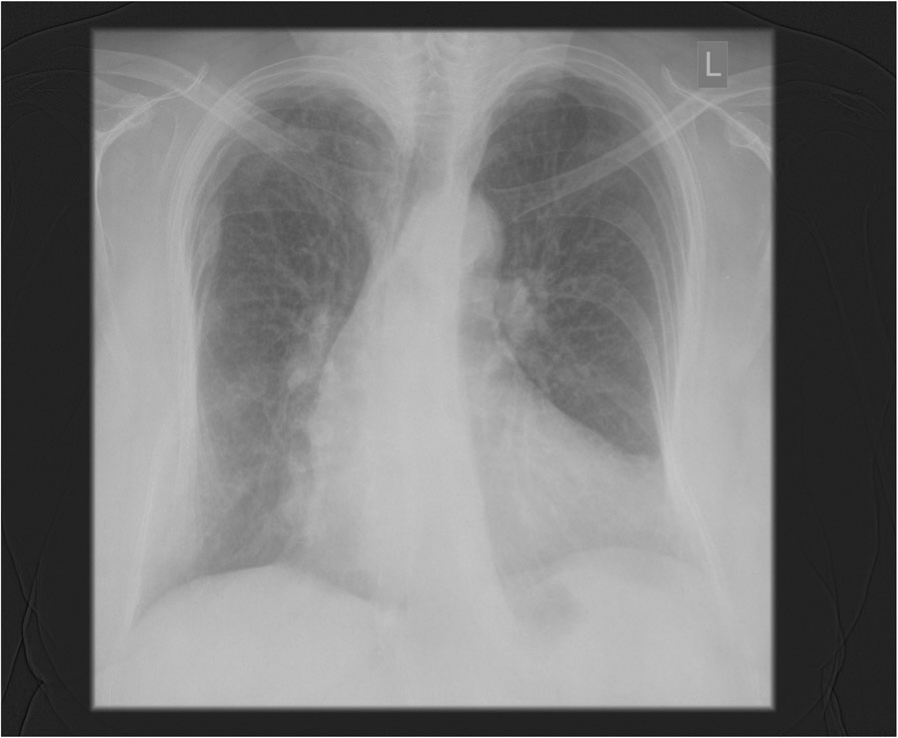
Chest X-ray (PA view)

**Fig. 2:**
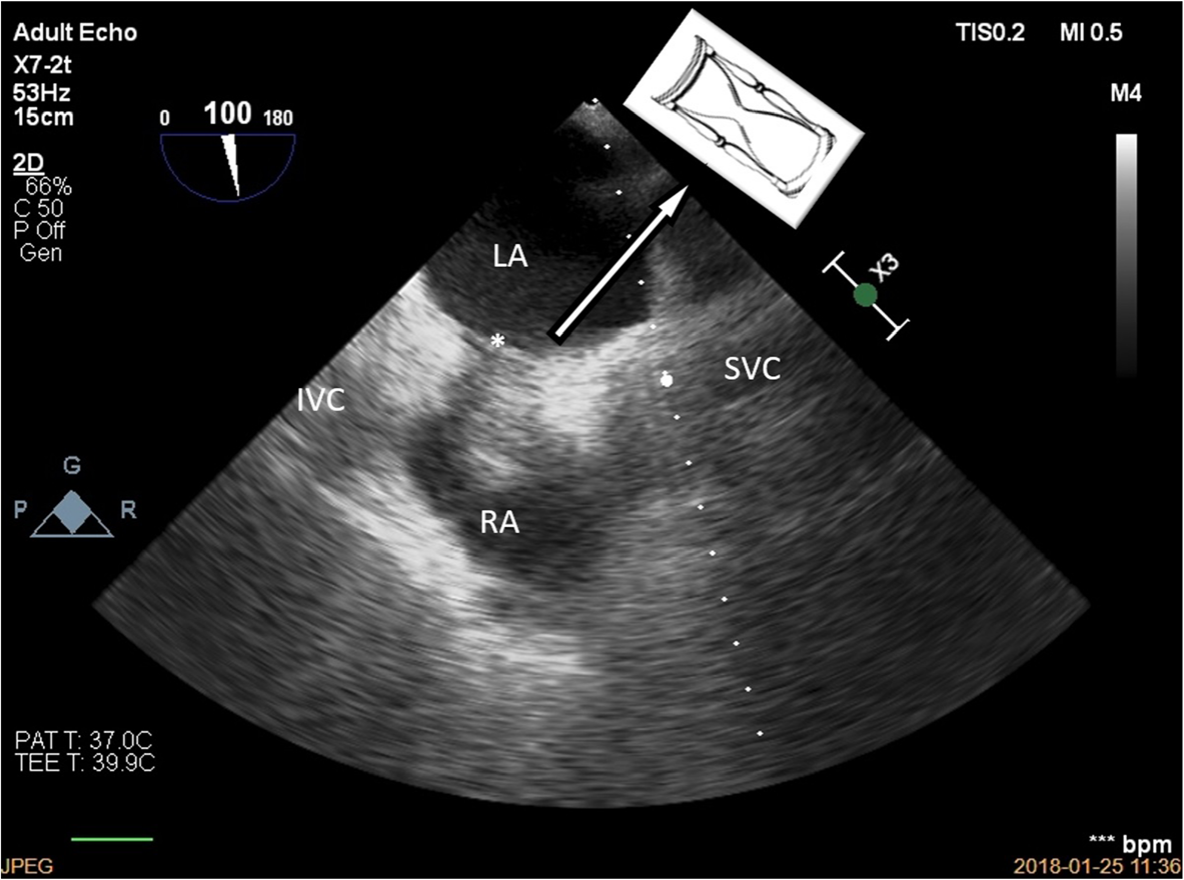
TEE. ME view at the level of fossa ovalis (*) demonstrates lipomatous tissue, with a characteristic hourglass (dumbbell) shape, infiltrating the septum between the right (RA) and left atria (LA) with sparing of the fossa ovalis. The change is located very close to the ostium of the inferior vena cava (IVC)

**Fig. 3:**
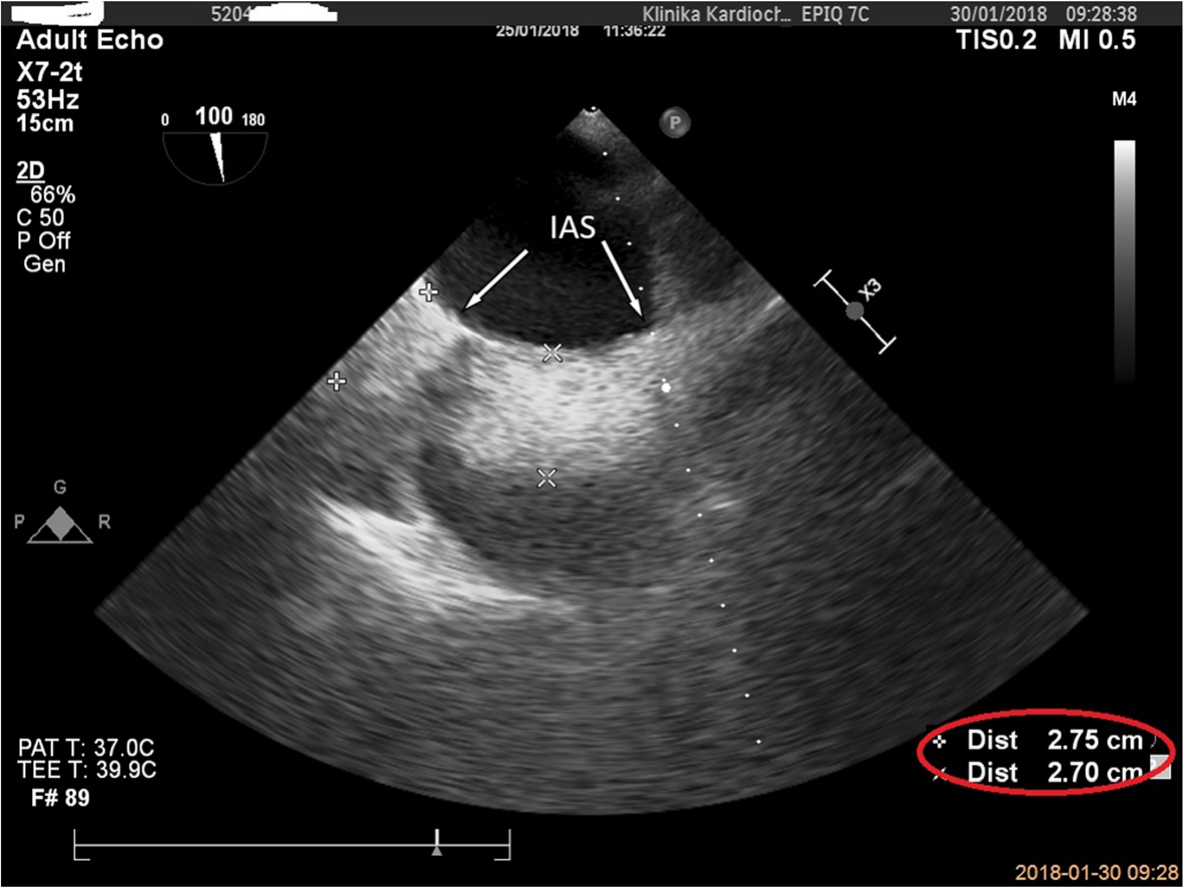
TEE. ME view demonstrates clearly visible boundaries of the interatrial septum (IAS) with intense hyperechogenic, measuring 2.75 cm and 2.7 cm, lipomatous tissue, respecting the fossa ovalis

We present a case of a 65-year-old female patient admitted to the Cardiac Surgery Department in Wroclaw in January 2018 with severe mitral regurgitation (MR) and the history of ischemic heart disease, after elective percutaneous coronary intervention of the circumflex branch of left coronary artery with two drug-eluting stents (DES) implantation 4 years earlier. Furthermore, the patient diagnosed with many chronic conditions, such as metabolic syndrome, obesity with BMI 33 and gastroesophageal reflux disease. Currently, with an exercise dyspnoea for about 2 years, intensifying in recent weeks, she was hospitalized in the Cardiology Department for further diagnostics. The transthoracic echocardiography (TTE) revealed non dilated left ventricle with a normal systolic ejection fraction of 60%, and no evidence of segmental wall motion abnormalities*,* severe MR with the prolapse of the A2 segment and systolic restriction of the posterior leaflet. Colour Doppler showed a highly distinctive eccentric turbulent jet directed towards the lateral wall and the base of the left atrium with ERO 0.6cm^2^ and regurgitant volume of 60 ml. Additionally, in the performed coronary angiography, hemodynamically significant narrowing was found in the area of the previously implanted DES. The patient was then consulted by the cardiac surgeon and qualified for surgery. After admission to the Cardiac Surgery Department, as part of the pre-operative preparation, TTE was again performed, in which the severe MR was confirmed and no pathological structures in the right atrium were described. Due to the unclear image in the right pulmonary field, described by the radiologist in the chest X-ray (Fig. 1), diagnostics was extended by performing a computed tomography of the chest, which excluded the presence of pathological shadow in the lung parenchyma. There was no referral to the atrial septum in the CT report. The patient was scheduled for mitral valve repair surgery and coronary artery bypass grafting (CABG) with the use of saphenous vein graft to the circumflex artery. During the standard procedure of commencing the cardiopulmonary bypass (CPB) and bicaval cannulation, it was found difficult to insert the cannulas from the atrium into both vena cavas. Therefore the cannulation was performed using the smaller cannula sizes, which eventually allowed to go on bypass. On the free wall of the atrial septum, there was a thickening and an excess of adipose tissue with a firm consistency and the size of a walnut, significantly impeding access to the operated mitral valve through the left atrium, and probably completely preventing surgery by the transseptal approach. In the transesophageal echocardiography (TEE), a characteristic image of LHAS was confirmed by the presence of hypertrophy of the septum, up to 2.7 cm, an hourglass shape with a characteristic indentation at the place of the fossa ovalis (Figs. 2 and 3). Based on the intra-operative TEE, as well as by analyzing the chest X-ray and CT scans, the diagnosis of LHAS was made. Due to the asymptomatic course of the LHAS and the complexity of the scheduled operation, the decision was made to leave the change intact. The mitral valve was replaced through the left atrial approach. The surgery was completed in a standard manner and the weaning from the CBP went uneventfully. The patient’s early postoperative period was a routine.

## Discussion and conclusions

Lipomatous hypertrophy of the atrial septum is a rare but increasingly recognized non-neoplastic benign abnormality of the heart. Since the first mention in literature in 1964, fewer than 300 cases of LHAS have been described, most of which were based on autopsy studies. Lipomatous lesion derives entirely from the upper and/or lower part of the atrial septum, typically sparing the fossa ovalis, giving a characteristic, considered by some to be pathognomonic, an hourglass-shaped image, with a tendency to bulge into the right atrium, which may be related with a thickening of crista terminalis (Fig. 3). The first descriptions of LHAS in vivo were made on the basis of echocardiography, and in 1983 Fyke et al. published the first diagnostic guidelines [16]. Currently, TTE, TEE, CT (preferred multislice CT - MSCT) and MRI are used for diagnostics. Transthoracic echocardiography, usually performed first, is not a very accurate diagnostic tool with limited imaging of the heart structures, however, transesophageal examination (TEE) is much more precise and shows the pathological mass quite well. CT scans enable the visualization of adipose tissue. Lipomatous changes also demonstrate minimal contrast enhancement, which allows us to exclude the other suspected pathologies. The typical localization, shape, and image, including the density of changes in CT, allows to differentiate LHAS from heart tumors and make a diagnosis without first confirming in histopathological examination [8, 17]. The actual incidence of LHAS in the population is not known. This is due to its asymptomatic course, and the lack of well-targeted diagnostics. Previously described in the literature cases were based on autopsy, surgery and clinical imaging incidental findings or were associated with the symptomatic course of the disease. Among the rare risk factors of LHAS are emphysema with steroid therapy, in which the predisposition to the mediastinal and intracardiac deposition of adipose tissue is observed, cereberotendinous xantomatosis, mediastino-abdominal lipomatosis and long-term parenteral nutrition [1, 11]. Arrhythmia, rarely associated with LHAS, was first observed in 1969 by Kluge [18]. The mechanism of its formation has not been explained, however, it seems to be related with the infiltration of adipocytes interfering with the structure of the atrial myocytes, and thus the normal conduction pathways are interrupted [11, 19]. Arrhythmia manifests mainly in atrial fibrillation, atrial premature complexes, supraventricular arrhythmias, ectopic and junctional rhythm. It is also presumed that the incidence of atrial arrhythmia is related to septal thickness [20]. Dickerson et al. presented a case of the patient with symptomatic LHAS who had atrial flutter, and following complete resection of the lesion, the symptoms completely resolved [21]. The mechanism in which the removal of LHAS resulted in the return of the sinus rhythm is unclear, although the authors speculate that the area of arrhythmogenic foci involved the right atrial wall with the crista terminalis, and as a result of resection, the pathological path between the superior vena cava (SVC) and the right atrium was discontinued, just like in the Cox-Maze procedure.

**Fig. 4:**
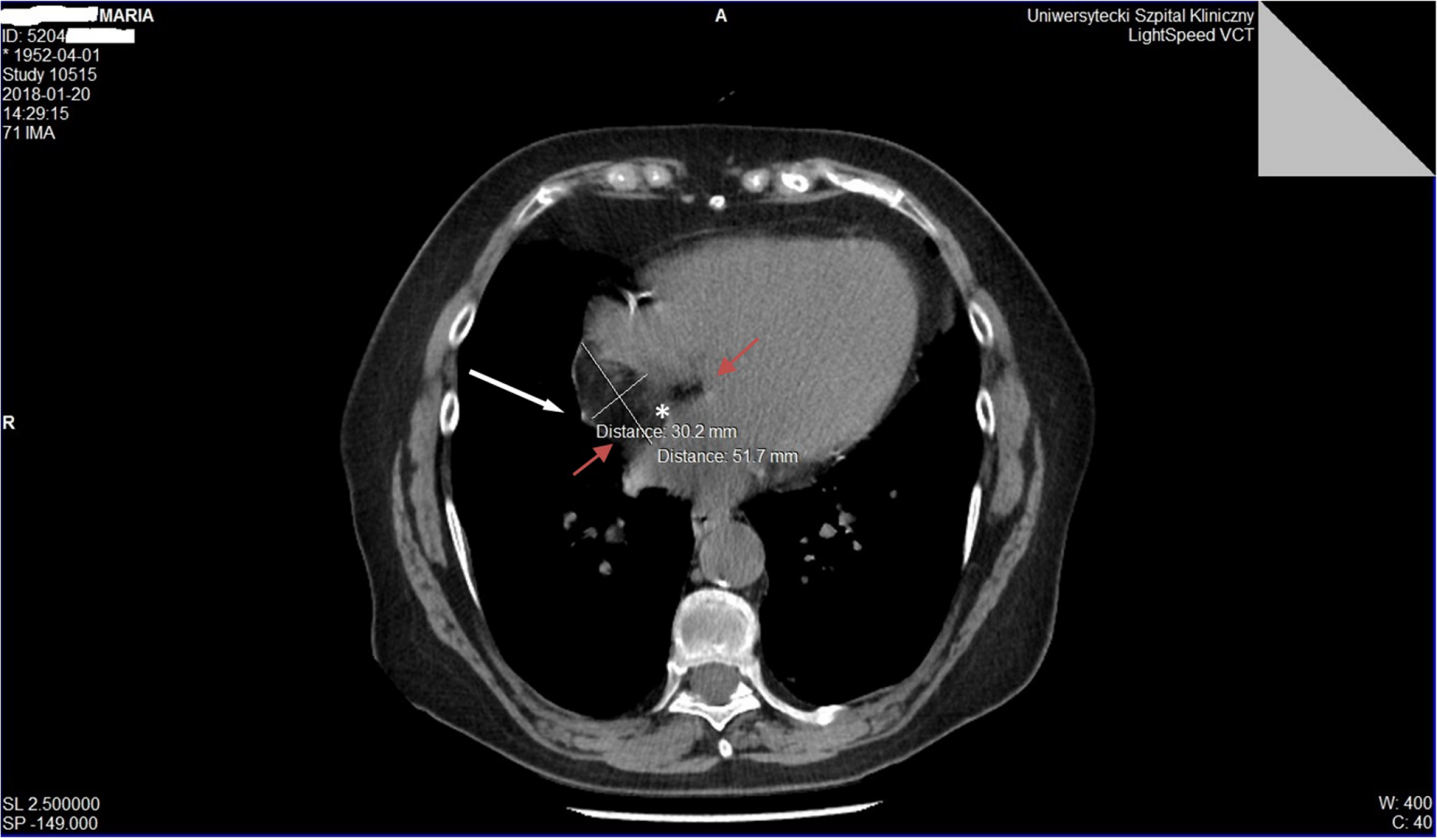
CT scan. Visible lipomatous tissue surrounding the fossa ovalis (*) with the characteristic hourglass shape (between the red arrows). The marked mass, measuring 30.2 mm × 51.7 mm, extends to the back wall of the right atrium (RA) and crista terminalis (white arrow)

**Fig. 5:**
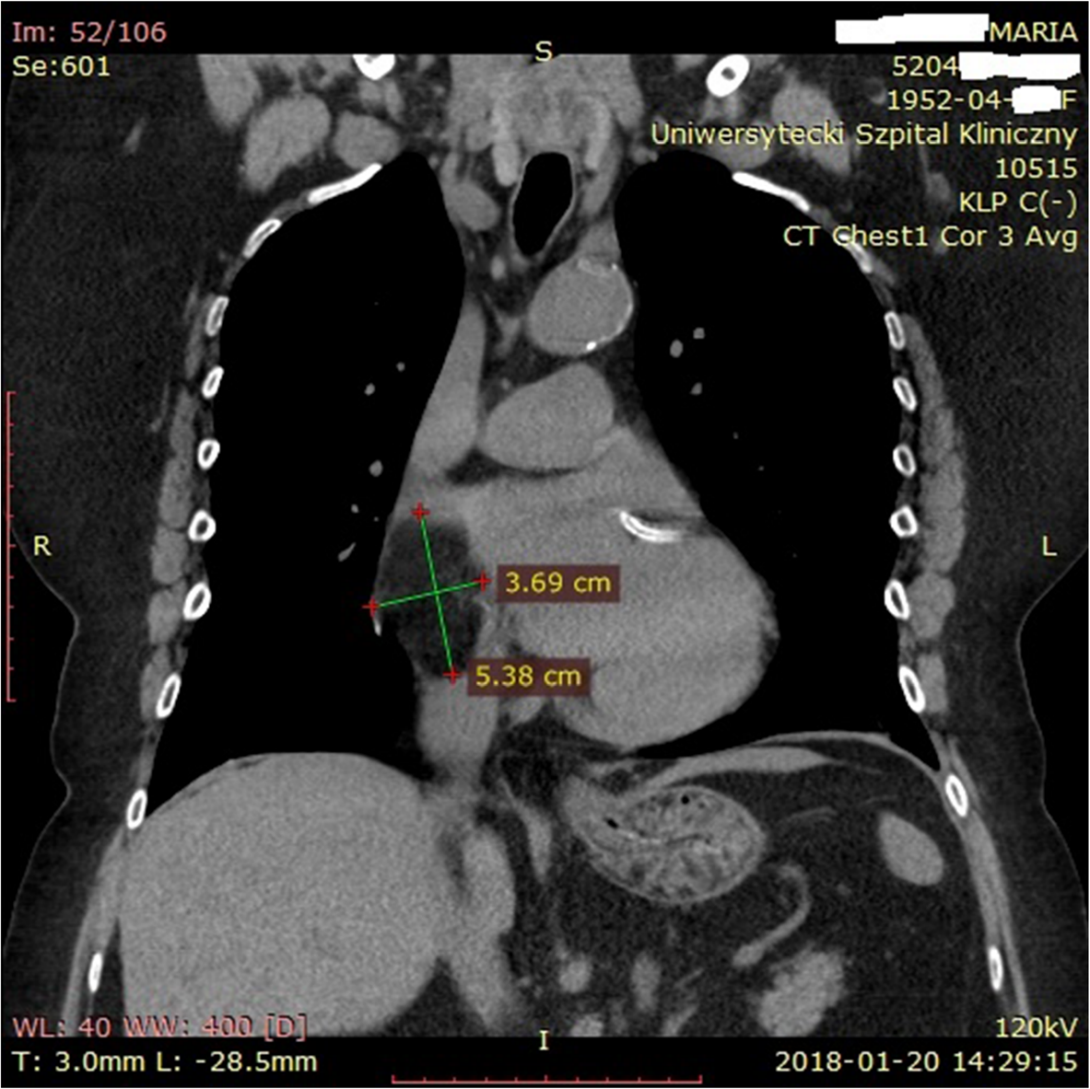
CT scan. Lipomatous mass in the interatrial septum (IVS) bulging to the right atrium (RA), in the axis of the SVC and IVC, measuring 3.69 cm × 5.38 cm

LHAS can cause undesirable consequences due to its size and localization. An abundant volume causing atrial septum bulging into the atrial cavities may cause symptoms, but most importantly may render some surgical and percutaneous interventions particularly challenging. Therefore, while pre-interventional recognition of LHAS in certainly important for cardiac surgery, this is even more important for invasive cardiological interventions involving transseptal catheterization access. This approach is commonly used in interventional cardiology, to treat number of anatomical defects of the heart, such as closure of atrial septal defects (ASDs), patent foramen ovales (PFO) or correction of the functional mitral regurgitation through percutaneous “edge-to-edge” mitral valve repair, as well as in interventional electrophysiology to treat left atrial arrhythmias through commonly used transseptal puncture [6]. A very rare problem related to the size of the mass and the anatomy of the right atrium are technical difficulties, as described above, during the bicaval cannulation, when commencing the CPB and difficulties in accessing the operated heart structures. In the presented case, resistance was encountered while inserting the venous cannulas. Based on the standardly performed intraoperatively TEE, as well as by analyzing the chest X-ray (Fig. 1) and CT scans, a diagnosis of LHAS was made (Figs. 4 and 5). Choosing the smaller cannula sizes allowed for an effective cannulation and transition to cardiopulmonary bypass in order to perform the surgery. Access from the left atrium to the operated mitral valve was significantly impeded, and the transseptal approach, without disturbing the LHAS structure, could not be possible.

In the presented case, a complex cardiac surgery was successfully performed. Asymptomatic LHAS does not require cardiac surgery. Surgical treatment of LHAS should be limited only to cases of patients with marginal obstruction of the SVC or the right atrium, which is an indication for a resection of the lesion with simultaneous interatrial septum plasty. The performed procedure may be a beneficial therapeutic option in patients with arrhythmia [1, 9, 21]. Long-term benefits of the surgery and the risk of recurrence have not been investigated [2].

## Abbreviations

BMI: Body mass index
CABG: Coronary artery bypass grafting
CPB: Cardiopulmonary bypass
CT: Computed tomography
DES: Drug-eluting stents
ECG: Electrocardiogram
ERO: Effective regurgitant orfice
IAS: Interatrial septum
IVC: Inferior vena cava
LA: Left atrium
LHAS: Lipomatous hypertrophy of the atrial septum
MR: Mitral regurgitation
MRI: Magnetic resonance imaging
MSCT: Multislice computered tomography
RA: Right atrium
SVC: Superior vena cava
TEE: Transesophageal echocardiogram
TTE: Transthoracic echocardiography
